# Baltimore College of Dental Surgery

**Published:** 1872-03

**Authors:** 


### ARTICLE IV.

### *Baltimore College of Dental Surgery*

The *Thirty-second* Annual Commencement of this College was held in Concordia Opera House, on Thursday evening, February 29th, in the presence of a very large audience.

The stage was occupied by the Faculty of the College, the Graduating Class, and a number of visitors, among the latter being Dr. Wayt, of Richmond, Va., one of the oldest and most eminent practitioners of the South, and whose youngest son was one of the graduates, the eldest having graduated in 1855.

After the rendering of several selections of music by the Harmonia Band, the exercises commenced with prayer by the Rev. Dr. E. R. Eschbach, which was followed by the Dean announcing the names of the graduates, and also the authority by which the degree was conferred upon them.

The degree of D.D.S., was then conferred upon the following gentlemen by Professor F. J. S. Gorgas, Dean of the Faculty:

| *Graduates:* | *Subject of Thesis:* |
|---|---|
| Robert Benjamin Adair. *Ga.* | Extraction of Teeth. |
| Francis Taliaferro Allen, *Va.* | The Arrest of Caries. |
| Benson Boggs Bushfield, *W. Va.* | Dental Caries. |
| Peter Abraham Dantzler, *S. C.* | Dental Prosthesis. |
| Tilghman Howard Ferguson, *Cal.* | Teeth and their Treatment. |
| James Lawrence Fogg, *Ga.* | Development of the Teeth. |
| Finis Ewing Gaston, *Miss.* | Insertion of Art. Dentures. |
| James Frank Griffith, *N. C.* | Anæsthetic Agents. |
| George Arthur Hildebrand, *N. C.* | Formation of the Teeth. |
| Bethel McMullen, *Fla.* | Odontalgia. |
| Henry Clay Moseley, *Texas.* | Alveolar Abscess. |
| Hugh Thompson Murray, *D. C.* | Sensitive Dentine. |
| Bernhard Myer, *Md.* | Tic Douloureux. |
| James Henry Nash, *Miss.* | Impression Materials. |
| Walter Robert Snead, *N. C.* | Nitrous Oxide Gas. |
| William Roscoe Turk, *Va.* | Digestion. |
| Charles Neville Wallach, *D. C.* | Materials for Filling Teeth. |
| George Greenhow Wayt, M.D. *Va.* | Causes & Treatment of Caries. |

Every one of the class on receiving his diploma, was the recepient of floral favors from his fair friends among the audience. After the conferring of the degrees, Professor P. H. Austen delivered the address to the graduates, giving them excellent advice and reminding them that their future success rested in a great degree with themselves. He urged upon them the importance of prosecuting dentistry as an art, and not solely as a means to enrich themselves; to avoid the temptations of the present age, which he characterized as the "golden;" to regard with horror as well as sorrow the spectacle of corruption on all sides; also the importance of of a classical education. At the close of his address, the speaker was warmly applauded. An address on behalf of the graduating class was then delivered by T. H. Ferguson, of California, who expressed the thanks of himself and class-mates for the benefits conferred upon them by the College, their appreciation of the kindness of the Faculty, and their gratitude and delight at the presence of such a large audience. He also referred, in a humorous manner, to the means presented by the press, large staring signs, marble figures, &c., for bringing their names and calling before the public, and concluded by predicting for those who pursued an honest and virtuous course, a successful future. The exercises ended with music and the benediction. The addresses delivered during the evening will appear in the next number of the *Journal.* We cannot close without referring, briefly, to the history of this institution, the oldest in the world, and one which has taken the lead in this branch of special surgery not only in America but in Europe also. The record of the Baltimore College of Dental Surgery shows, that since it was chartered in 1839, by the General Assembly of Maryland, 682 students have received its diploma, and during the last twenty years, dating only as far back as 1852, nearly 1,000 students have been in attendance upon the lectures. There is a Dispensary attached to the College, at which a large amount of charitable work is done for the inmates of orphan asylums, patients of the various dispensaries throughout the city and other charitable institutions.

Though so old an institution and doing so much charitable work, this College has never received any aid from either the city of Baltimore or the State of Maryland.

We understand a bill is now before the Legislature asking for an appropriation to establish a large "Free Infirmary," with all the necessary dental and surgical appliances for a complete and thorough establishment. The object is to have this Dental Infirmary the clinical lecture room of the College, and to bear the same relation to the dental school that the hospital does to the medical; it will in fact be a necessary appendage to such a school, as it will undoubtedly add greatly to the practical knowledge and efficiency of the graduates.

For many years the Baltimore College was the only dental institution in the world; now, however, there are nine Dental Colleges in this country.

